# Decreased anticipated pleasure correlates with increased salience network resting state functional connectivity in adolescents with depressive symptomatology

**DOI:** 10.1016/j.jpsychires.2016.07.013

**Published:** 2016-11

**Authors:** Ewelina Rzepa, Ciara McCabe

**Affiliations:** School of Psychology and Clinical Language Sciences, University of Reading, UK

**Keywords:** fMRI, Depression, Biomarker, Resting-state, Connectivity, Salience network, Adolescent, DMN

## Abstract

Previous studies have found dysfunctional resting state functional connectivity (RSFC) in depressed patients. Examining RSFC might aid biomarker discovery for depression. However RSFC in young people at risk of depression has yet to be examined.

35 healthy adolescents (13–18 yrs old.) were recruited. 17 scoring high on the Mood and Feelings Questionnaire (MFQ > 27 (High Risk: HR), and 18 scoring low on the MFQ < 15 (Low Risk: LR) matched on age and gender. We selected seed regions in the salience network (SN: amygdala and pregenual anterior cingulate cortex (pgACC)) and the central executive network (CEN: dorsal medial prefrontal cortex (dmPFC)). Mood and anhedonia measures were correlated with brain connectivity.

We found decreased RSFC in the HR group between the amygdala and the pgACC and hippocampus and precuneus. We also found decreased RSFC in the HR group between the pgACC and the putamen and between the dmPFC and the precuneus. The pgACC RSFC with the insula/orbitofrontal cortex correlated inversely with the anticipation of pleasure in all subjects. Increased RSFC was observed between the pgACC and the prefrontal cortex and the amygdala and the temporal pole in the HR group compared to the LR group.

Our findings are the first to show that adolescents with depression symptoms have dysfunctional RSFC between seeds in the SN and CEN with nodes in the Default Mode Network. As increased connectivity between the pgACC and the insula correlated with decreased ability to anticipate pleasure, we suggest this might be mechanism underlying the risk of experiencing anhedonia, a suggested biomarker for depression.

## Highlights

• This is the first study to examine RSFC in healthy adolescents with increased depression symptomatology.
• We find RSFC between the pgACC and the insula/OFC correlates with ability to anticipate pleasure.
• We suggest neural mechanisms for risk for clinical depression.

## 1 Introduction

Adolescence is a crucial developmental period where the incidence of depression increases significantly, and reports have emphasised that around 8% of adolescents are affected by depression by the age of 16 (Saluja et al., 2004). Identifying biomarkers such as dysfunctional neural networks could help develop preventative treatments for young people at increased risk of clinical depression.

It is been shown that patients with major depressive disorder (MDD) have abnormalities in their resting state functional connectivity (RSFC) in networks such as the Salience Network (SN) the Central Executive Network (CEN) and the Default Mode Network (DMN) (Sheline et al., 2010).

The Salience Network (SN) and the central executive network (CEN) show strong task-related activation and are less active at rest in healthy controls. The SN which consists of regions such as the anterior insula, pregenual anterior cingulate and amygdala are implicated in the processing of various aspects of salient stimuli whereas the CEN, which consists of regions such as the dorsolateral, dorsal medial prefrontal cortex and the posterior parietal cortex, is involved in cognitive functioning including attention and working memory (Bressler and Menon, 2010). In MDD, abnormalities in these networks are thought to reflect deficits in attentional control over emotional stimuli, difficulties with suppression of unwanted thoughts and difficulties with emotion recognition. However there have been inconsistencies in the direction of effects with some studies finding increased SN and CEN (Horn et al., 2010, Manoliu et al., 2014, Ramasubbu et al., 2014, Sheline et al., 2010), whilst others find reduced connectivity (Liston et al., 2014, Tahmasian et al., 2013, Ye et al., 2012) in these regions in MDD. These inconsistencies might be related to differences in the MDD population studied (adults, elderly, adolescents), medication history and depression severity.

There is small number of RSFC studies in adolescents with MDD. These studies have mostly focused on investigating the SN and they report decreased RSFC between the amygdala and the hippocampus, parahippocampus and brain stem which has also been shown to correlate with depression severity (Cullen et al., 2014). The same study however, also showed increased RSFC between the amygdala and the precuneus in depressed adolescents. Mixed results were also reported by Pannekoek et al. who found both increased RSFC between the amygdala and the parietal cortex in MDD adolescents and decreased RSFC between the amygdala and regions such as the pgACC, frontal pole and the paracingulate gyrus (Pannekoek et al., 2014).

The authors suggested that risk for depression may be related to dysfunction in brain areas involved in supporting self-relational processes and reward prediction. Another study examining familial risk for depression in adolescents found decreased RSFC between the prefrontal cortex and parts of the CEN, in those with a parent with depression which also correlated the parents depression severity (Clasen et al., 2014). The authors suggested that an increase in vulnerability to depression may thus be underpinned by altered development of the CEN in young people at risk.

Our current study aims to examine RSFC in another at risk group, adolescents with increased depression symptomatology but with no clinical diagnoses. Based on the previous literature, we selected seed regions that have been shown dysfunctional in depressed patients and adolescents at familial risk of depression, amygdala and pgACC seeds from the SN and the dmPFC highlighted as a key node of within the CEN and dysfunctional RSFC in depressed patients (Sheline et al., 2010). We hypothesised that adolescents at risk of depression would also have decreased RSFC between key brain regions implicated in the aetiology of depression, supporting the notion that dysfunctional resting state neural networks may be biomarkers for depression.

## 2 Materials and methods

### 2.1 Participants

35 healthy adolescents (13–18 yrs old.) were recruited. 17 healthy adolescents scoring high on the Mood and Feelings Questionnaire (Costello et al., 1998) (MFQ > 27 (HR), and 18 adolescents scoring low on the MFQ < 15 (LR) matched on age and gender. Participants completed the MFQ; a self-report questionnaire designed to assess recent (last 2 weeks) presence and severity of depressive symptoms as specified in DSM-IV. The measure is composed of 33 statements corresponding to how often the individual has experienced particular behaviours and feelings during this time. There is considerable psychometric data for this child version, including good test–retest reliability for a score of 27 and above indicating increased depression symptom severity (Pearson's r ¼ 0.78) (Wood et al., 1995) and below 15 indicating healthy controls (Kyte et al., 2005).

Participants who scored between 15 and 27 were excluded from the study. The University of Reading Ethics Committee provided ethical approval and the investigation was carried out in accordance with the latest version of the Declaration of Helsinki. We obtained written informed consent from all participants before screening and after giving the complete description of the study. Exclusion criteria for all subjects consisted of current or past history of alcohol or drug dependency, pregnancy and any contradictions to MRI, e.g. pacemaker, mechanical heart valve, hip replacement, metal implants. Further, both of the groups were determined to be free from current or past axis 1 disorder (including anxiety disorders, depression, eating disorders psychosis and substance abuse) on the structured clinical interview for DMS-IV (Spitzer et al., 2004). None of the participant took current medication apart from the contraceptive pill. All subjects were rated on the following questionnaires: Mood and Feeling Questionnaire (MFQ; (Angold et al., 1995)), Beck Depression Inventory (BDI; (Beck et al., 1961)), the Fawcett–Clarke Pleasure Scale (FCPS; (Fawcett et al., 1983)), and the Snaith–Hamilton Pleasure Scale (SHAPS; (Snaith et al., 1995)), the Temporal Experience of Pleasure Scale, consummatory subscale TEPS-C and anticipatory subscale TEPS-A (Gard et al., 2006), before scanning. Body mass index (BMI) for each individual was also calculated.

### 2.2 Overall design

MRI derived measures of brain function, based on blood-oxygenation-level-dependent (BOLD) contrast were used to compare brain responses at rest across the LR group and the HR group. The resting-state data were acquired before any other scans including the structural scan. Subjects were instructed to lie in dimmed light with their eyes open, think of nothing in particular, and not to fall asleep, similar to our previous studies (Cowdrey et al., 2012, McCabe and Mishor, 2011, McCabe et al., 2010, Rzepa et al., 2015) and a method found to have higher reliability than eyes closed (Patriat et al., 2013). To measure whether undergoing a scan had an effect on mood change, volunteers completed the Befindlischkeit scale on mood (BFS; (Hobi, 1985)) and an energy and affect Visual Analogue Scale (VAS) pre and post scan.

### 2.3 Image acquisition

A Siemens Magnetom Trio 3T whole body MRI scanner and a thirty-two-channel head coil were used. Multi-band accelerated echo planar imaging sequencing (Center for Magnetic Resonance Research, Minnesota) was used with an acceleration factor of 6 and iPAT acceleration factor of 2. T2*-weighted EPI slices were obtained every 0.7 s (TR = 0.7, TE = 0.03), these parameters were optimised given our scanner capability and used to increase sampling rates and increase our power to detect resting state networks as has been shown previously with multiband (Xu et al., 2013, Filippini et al., 2014). 54 transverse slices with in-plane resolution of 2.4 × 2.4 mm were attained and slice thickness was 2.4 mm. The matrix size was 96 × 96 and the field of view (FOV) were 230 × 230 mm. Acquisition was performed during resting-state scan, yielding 400 vol in total. Sagittal 3D MPRAGE images were also acquired with an isotropic in-plane resolution of 1 × 1 × 1 (TI = 0.9 s, TR = 2.02, flip angle 9°, FOV = 250 × 250 mm) yielding 192 slices.

### 2.4 fMRI data analysis

#### 2.4.1 Pre-processing

Imaging data were pre-processed and analyzed using FSL tools (www.fmrib.ox.ac.uk/fsl) (Smith et al., 2004). fMRI data pre-processing was carried out using FEAT (FMRI Expert Analysis Tool, Version 6.0, a part of FSL software), and included the following steps: non-brain removal (Smith, 2002), motion correction using MCFLIRT(Jenkinson and Smith, 2001), spatial smoothing using a Gaussian kernel of full-width at half maximum (FWHM) of 5 mm, grand mean intensity normalization of the entire 4D dataset by a single multiplicative factor and high pass temporal filtering **(**Gaussian-weighted least-squares straight line fitting, with sigma = 64.0s**).** fMRI volumes were registered to the individual's structural scan and the MNI-152 standard space image (Montreal Neurological Institute, Montreal, QC, Canada) using FMRIB's Linear Image Registration Tool (FLIRT).

#### 2.4.2 Time series extraction and higher level analysis

To study resting-state functional connectivity, a seed-based correlation approach was used. Using the Harvard-Oxford subcortical structural atlas (Kennedy et al., 1998) we created a structural bilateral amygdala seed as the amygdala is a small structure and not suitable for a ROI sphere. To maximize the exact coverage, the masks of these seed regions were threshold by 20% to include voxels having at least 80% of probability of being in these particular regions. We also created seeds for the dmPFC (18 34 29; −24 35 28) (6 mm sphere so as to not cross into other brain regions) coordinates from (Sheline et al., 2010) and pgACC (8 mm sphere with a center at 0 38 0 so as to not cross into other brain regions). The dmPFC and pgACC seeds were created with Wake Forest University Pickatlas tool in SPM8 as in our previous study and can be seen in the figures (McCabe et al., 2011).

The mean time course within the left and right seeds of each ROI (except for the pgACC, only comprising one medial seed) was calculated and used as a regressor in a general linear model. In addition, white matter signal, cerebrospinal fluid signal, 6 motion parameters (3 translations and 3 rotations), and the global signal were used as nuisance regressors. We have obtained white matter and cerebrospinal fluid masks using FSL's FAST segmentation program. The resulting segmented images were then thresholded to ensure 80% tissue type probability. For each individual, the general linear model was analyzed by using the FMRI Expert Analysis Tool [version 5.4, part of FMRIB's Software Library (Smith et al., 2004)]. The resulting parameter estimate maps were then analyzed using higher level 1 sample *t*-tests for group averages and between samples *t*-tests for group differences. Clusters were determined by Z > 2.3 voxel-wise thresholding and a family-wise error-corrected cluster significance threshold of P < 0.05 (Worsley, 2001). From the results we then only report those that met the further correction for number of ROIs examined which gave *P* < 0.016 (i.e, *P* < 0.05 Bonferroni corrected for the 3 networks of interest: amygdala, dmPFC, pgACC (Davidson et al., 2003). Next, the % BOLD signal change data was extracted from the regions of significant effect (Table 2) using the FSL tool Featquery (www.fmrib.ox.ac.uk/fsl) (Smith et al., 2004).

#### 2.4.3 Correlational analyses

To examine the relationship between the scores on behavioural questionnaires and RSFC we extracted the % BOLD signal change using FSL Featquery and correlated with scores on our questionnaires.

## 3 Results

### 3.1 Demographic and clinical data

Demographic data analysis (Table 1) revealed no significant age and gender differences between HR and the LR groups. Results for BMI scores were calculated and there was a significant BMI difference between the HR and the LR group. There were also significant differences between the two groups on measures of mood, depression and anhedonia (Table 1) (MFQ, BDI, SHAPS, FCPS, TEPS-A, TEPS-C) (Table 1).

### 3.2 Mood, energy and affect scores

Repeated measures ANOVA with within subject factor of time (before and after scan) and between subject factor of group (HR and LR) was employed to examine whether there would be any differences on scores of mood as measured by the BFS. Results for BFS revealed that there was no significant main effect of time (*F*(1.33) = 0.011; *p* = 0.919) but there was a significant main effect of group (*F*(1.33) = 11.33; *p* = 0.002) and significant interaction between time and group (*F*(1.33) = 5.56; *p* = 0.025). Further paired sample *t*-test analysis revealed that there was a significant difference for time in the LR group (*t*(17) = −3.08; *p* = 0.007) meaning they felt worse after the scan and non significant difference for time in the at HR group (*t*(16) = 1.29; *p* = 0.216) (Table S1).

Repeated measures ANOVA with within subject factor of time on two levels (before and after scan) and within subject factor of Emotion on nine levels (alertness, disgust, drowsiness, sadness, happiness, anxiety, withdrawn, faint, nausea) and between subject factor of group (healthy controls and at risk) was employed to examine whether there will be any differences between the LR and HR groups scores of energy and affect, as measured by VAS. Results for VAS revealed that there was no significant main effect of time (*F*(1.33) = 0.385; *p* = 0.539) and no significant main effect of group (*F*(1.33) = 3.37; *p* = 0.075) but there was a significant main effect of Emotion (*F*(8.264) = 57.78; *p* < 0.001) yet no significant interaction between the time, emotion and group (*F*(8.264) = 1.28; *p* = 0.252). Further paired sample *t*-test analysis revealed that there was a significant difference for time in the LR group for disgust (*t*(17) = −2.949, *p* = 0.009) feeling faint (*t*(17) = −2.164, *p* = 0.045) and in the HR group for drowsiness (*t*(16) = 2.57; *p* = 0.02) and anxiety (*t*(16) = 2.14; *p* = 0.049) all increasing after the scan except anxiety (Table S1).

### 3.3 Main effects of stimuli on blood oxygen level-dependent responses

Table S2 provides a summary of the main effects, i.e. the brain regions that had RSFC with the seed regions (baseline) for the LR group only. Overall, the patterns of connectivity associated with each of the seed regions are consistent with resting-state and functional connectivity experiments in healthy controls, subjects at risk for depression and depressed patients (Bebko et al., 2015, Cullen et al., 2014, Guo et al., 2015, Sheline et al., 2009, Sheline et al., 2010, Shen et al., 2015, Clasen et al., 2014)

### 3.4 Effects of mood on RSFC

There was no main effect of age, gender, BMI or MFQ on RSFC.

### 3.5 Decreased functional connectivity in the HR group

#### 3.5.1 Left amygdala seed

**Fig. 1:**
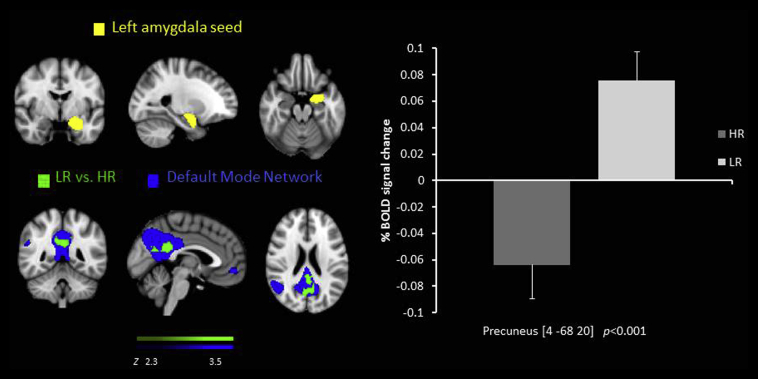
Resting state functional connectivity between the left amygdala seed region  and the precuneus, lower in the HR group than the LR  overlaid on the DMN . % BOLD signal change extracted for connectivity for both of the groups.

There was decreased RSFC in the HR group compared to the LR group between the left amygdala seed and the pgACC and the precuneus (Fig. 1) and posterior cingulate cortex (PCC) (Table 2).

#### 3.5.2 pgACC seed

There was decreased RSFC in the HR group compared to the LR group between the pgACC seed and the thalamus, palladium and the putamen (Table 2).

#### 3.5.3 Right amygdala seed

There was decreased RSFC in the HR group compared to the LR group between the right amygdala seed and the hippocampus (Table 2).

#### 3.5.4 Right dmPFC seed

**Fig. 2:**
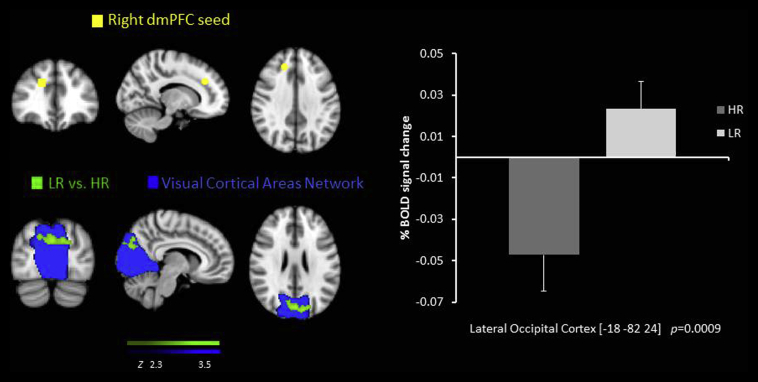
Resting state functional connectivity between the right dmPFC seed region  and the lateral occipital cortex, lower in the HR group than the LR  overlaid on the Visual Cortical Network . % BOLD signal change extracted for both of the groups.

There was decreased RSFC in the HR group compared to the LR group between the right dmPFC seed and the precuneus/cuneal cortex and the lateral occipital cortex (Table 2, Fig. 2).

### 3.6 Correlational analysis with behaviour

**Fig. 3:**
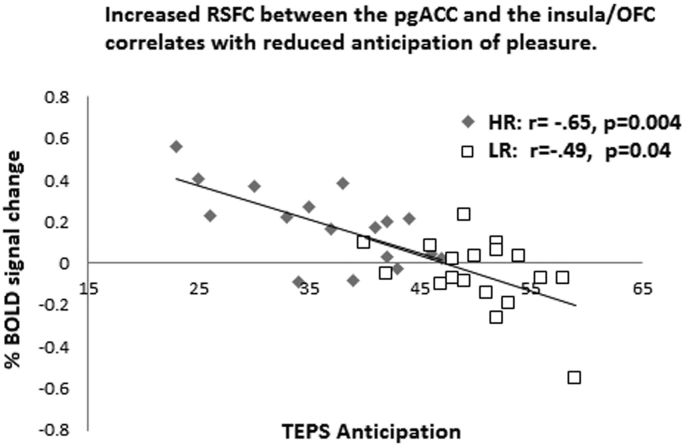
Increased resting state functional connectivity between the pgACC seed region and the insula/LOFC correlates with reduced anticipation of pleasure.

There was a negative correlation between increased RSFC of the pgACC seed and the insula/OFC and decreased anticipation of pleasure (TEPS-A) in both the HR (*r* = −0.65, *p* = 0.004) and LR groups (*r* = −0.48, *p* = 0.04) (Fig. 3).

### 3.7 Increased functional connectivity in the HR group

#### 3.7.1 Left amygdala seed

There was increased RSFC in the HR group compared to the LR group between the left amygdala seed and the temporal pole (Table 2).

#### 3.7.2 pgACC seed

**Fig. 4:**
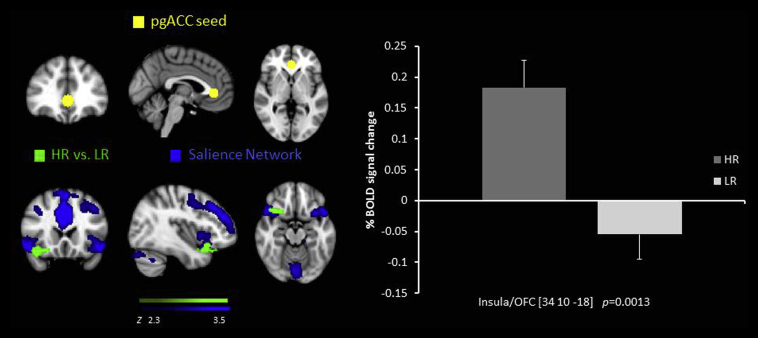
Resting state functional connectivity between the pgACC seed region  and the insula/LOFC, higher in the HR group than the LR  overlaid on the Salience Network . % BOLD signal change extracted for connectivity for both of the groups.

There was increased RSFC in the HR group compared to the LR group between the pgACC seed and the brain stem, the anterior cingulate cortex, the ventral medial prefrontal cortex and the OFC/insula (Table 2, Fig. 4).

All *p*-values for clusters were firstly determined by Z > 2.3 voxel-wise thresholding and a family-wise error-corrected cluster significance threshold of *P* < 0.05, then further Bonferroni corrected for number of ROIs examined which gave *P* < 0.016 (i.e, *P* < 0.05 (Davidson et al., 2003). OFC-orbitofrontal cortex, pgACC- pregenual anterior cingulate cortex, ACC-anterior cingulate cortex.

All data except * remained significant even when the global signal was not used as a nuisance regressor.

## 4 Discussion

The main aim of our study was to investigate RSFC in young people at risk of developing major depressive disorder by virtue of having increased depression symptomatology. We hypothesised deceased RSFC in key regions that have been found decreased in adults with depression such as regions in the SN and CEN.

Specifically we found decreased RSFC in the HR group compared to the LR group between the amygdala seed and the pgACC. The pgACC is claimed to be a node of communicating between the dorsal ACC important for error detection and attention and the more ventral ACC implicated in emotion processing, regulation and salience detection (Ball et al., 2014). Further, studies in depressed patients, remitted depressed patients and young people with depression symptoms find dysfunctional ACC activity during tasks involved in processing emotions and rewards (Ho et al., 2014, Fitzgerald et al., 2008, McCabe et al., 2009; Rzepa et al., in press), and recently in a RSFC study in depressed adolescents (Pannekoek et al., 2014). Thus it has been suggested that reduced RSFC between the amygdala and the pgACC may reflect problems in integrating inputs of positive information and influencing affect regulation, resulting in decreased hedonic responses and increased feelings of negativity. We found decreased RSFC between the pgACC seed and the thalamus, palladium and the putamen. These are areas of the brain involved in reward and emotion processing and also found dysfunctional in depression (Alexander et al., 1990, Phillips et al., 2003). As our results are in line with the decreased pgACC to amygdala, pallidum and thalamus RSFC found in depressed patients (Anand et al., 2005) it suggests that dysfunctional cortico-limbic connectivity in young people at risk of depression might be a biomarker underpinning problems with the control and regulation of emotional processes.

We also found decreased RSFC in the HR group compared to the LR group between the amygdala seed region and the PCC and the precuneus which have been implicated in self-referential processing in fMRI tasks and thought to underlie maladaptive rumination in depression (Nejad et al., 2013). Further these regions are key nodes of the default mode Network (DMN). The DMN is a network of distributed brain regions that show prominent activation during rest, and deactivation during the performance of cognitive tasks (Whitfield-Gabrieli and Ford, 2012). Studies have revealed that in healthy subjects, the DMN is associated with rumination and self-reflection and that greater suppression of DMN is related to a better performance on attention demanding tasks. Whilst some studies find the DMN overactive in MDD patients when compared with healthy controls, which has been suggested to underlie the symptom of negative rumination in depression (Whitfield-Gabrieli and Ford, 2012) others find decreased connectivity between the DMN and the ventral striatum and the sensorimotor cortex which also correlate negatively with behavioural inhibition in young people at increased familial risk of depression (Frost Bellgowan et al., 2015). However, reports of decreased amygdala RSFC with the precuneus in children at familial risk for depression and in depressed adolescents have been shown (Luking et al., 2011). Our results are in line with Luking at al. and suggest, that if adolescents with increased risk of depression have difficulty in emotion regulation this may be due to decreased amygdala-precuneus connectivity. To further explore this, future studies should examine the relationship between emotion processing and RSFC in young people at risk of depression.

We also found decreased RSFC in the HR group compared to the LR group between the amygdala seed and the hippocampus, similar to that found previously in MDD adolescents and in children with a family history of depression (Cullen et al., 2014, Luking et al., 2011). The amygdala possesses strong connections with the hippocampus which plays a crucial role in encoding and retrieval of emotional stimuli (Smith et al., 2006). Thus it could be suggested that decreased connectivity between the amygdala and the hippocampus might lead to less ability to suppress negative memories which in turn could be a risk factor for developing depression.

Our results also revealed decreased RSFC between the dmPFC seed and the precuneus part of the DMN and the lateral occipital cortex part of the visual network. The dmPFC is a key node in the CEN and is a structure implicated in many cognitive and emotional processes (Janssen et al., 2015, Jaworska et al., 2014). Similarly, recent studies have shown that RSFC between the CEN and the DMN is decreased in MDD (Abbott et al., 2013, Manoliu et al., 2013, Sheline et al., 2010) which has also been associated with patients’ difficulties to disengage from self-referential processes that may lead to negative thoughts (Manoliu et al., 2013). Thus our results are an extension of this, as we also see a similar pattern in young people at high risk but not currently diagnosed. Further, the decreased connectivity between the dmPFC and the occipital cortex in the HR group, found in our study, may indicate a mechanism by which compromised control over emotional images in adolescents could increase the risk of depression.

Interestingly, we also found *increased* RSFC between the pgACC seed and the insula, brain stem and frontal regions such as the ACC, vmPFC, lateral OFC and the amygdala seed and the temporal pole. As described above the pgACC is a key node for emotion processing, regulation and salience detection (Ball et al., 2014) and a previous study by Horn et al. also found altered pgACC RSFC with the anterior insula which was also related to depression severity and glutamate levels (Horn et al., 2010). The insula is thought to be an integration center for emotion, visceromotor, autonomic and interoceptive information and is also key node in switching between the CEN and the DMN during task performance (Guo et al., 2015, Sridharan et al., 2008). Given that we found that increased connectivity between the pgACC and the insula correlated with decreased ability to anticipate pleasure, we suggest this might be mechanism underlying the experience of anhedonia and therefore a possible further biomarker for depression in adolescents.

Increased RSFC between the amygdala and the temporal pole in the HR group may also be important given that the temporal pole has been implicated in studies examining Theory of Mind (the ability to predict other people's behaviour by attributing mental states such as believes and desires). For example a previous study in MDD patients reported increased amygdala to Temporal pole RSFC which also correlated inversely with depression severity (Ramasubbu et al., 2014). Interestingly, the authors argued that because the connectivity between these regions increased as depression severity decreased this may represent a compensatory mechanism by which subjects are more likely to maintain a balance in processing socially and emotionally relevant information. Intriguingly finding a similar pattern in our sample of young people at risk might also be related to resilience and protection against future depression development. Thus future studies would benefit from larger sample sizes and longitudinal designs to address this directly. Furthermore, our analysis examined the entire amygdala, but prior work has shown that amygdala subregions have known dissociable functional networks (Roy et al., 2009). Therefore future research should investigate how RSFC patterns in adolescents at risk of depression vary across amygdala subregions; interestingly such research would also benefit from the implementation of multiband sequencing that we have used in this study (Ugurbil et al., 2013).

Taken together we have shown that even in young people who are not currently depressed but who are at risk, due to depression symptomatology, there are decreased RSFC between key regions involved in the processing of salient stimuli and decision making. Further increased connectivity between amygdala and the temporal pole may also be an indicator of resilience to clinical depression in the future, we believe this is certainly worth investigating further.

## Conflict of interest

The authors have no conflict of interest.

## Author contributions

C McCabe and E Rzepa declare having materially participated in the research and/or article preparation. C McCabe and E Rzepa collected the data and analyzed the data and wrote the paper. Both authors have approved the final article.

## Funding and source

Funded by the University of Reading. The University had no role in collecting, analysing of writing up of the data.

## Figures and Tables

**Table 1:** Demographic and clinical characteristics.

Measure	HR (*n* = 17) mean (SD)	LR (*n* = 18) mean (SD)	*p*-value
Age (years)	16.59 (1.18)	16.33 (1.6)	0.598
Gender (male)	4/13	6/12	0.535
BMI	21.82 (2.72)	20.1 (1.94)	0.041
MFQ	40.41 (6.1)	4.4 (5.1)	<0.001
BDI	29.82 (12.7)	2.28 (4.13)	<0.001
FCPS	121.12 (18.7)	137.89 (21.3)	0.019
SHAPS	30.11 (5.56)	20.77 (8)	<0.001
TEPS-A	37.29 (7.58)	50.66 (5.14)	<0.001
TEPS-C	31.76 (5.98)	36.66 (7.28)	<0.001

**Table 2:** RSFC between seed regions and whole brain compared between groups.

Brain region	MNI coordinates			z-score	Cluster size	p-value
	X	Y	Z			
*LR* *>* *HR*						
***L Amygdala seed***						
pgACC	2	36	−2	6.13	4447	<0.001
Precuneus	4	−68	20	3.5	476	<0.001
PCC	0	−40	24	3.21	476	<0.001
***pgACC seed***						
Thalamus	0	−4	2	6.28	1150	<0.001
Pallidum	16	−4	−6	4.17	1150	<0.001*
Pallidum/Putamen	−16	6	−2	3.11	1150	<0.001
***R amygdala seed***						
Hippocampus	−14	−10	−18	4.05	357	0.00069
***R dmPFC seed***						
Cuneal Cortex/Precuneus	8	−76	36	3.15	401	0.0009
Lateral Occipital Cortex	−18	−82	24	3.1	401	0.0009
*HR* *>* *LR*						
***L Amygdala seed***						
Temporal pole	−46	4	−18	3.2	1473	<0.001
***pgACC seed***						
Brain stem	8	30	30	4.69	1156	<0.001
ACC	6	32	26	3.85	2552	<0.001
vmPFC	−6	50	−4	3.79	2552	<0.001
Lateral OFC	40	22	−16	3.57	369	0.0013
Insula/OFC	34	10	−18	3.27	369	0.0013
