# Supplementary material for: Decreased anticipated pleasure correlates with increased salience network resting state functional connectivity in adolescents with depressive symptomatology
Source: J Psychiatr Res. 2016 Nov;82:40–7. doi: 10.1016/j.jpsychires.2016.07.013 (PMC5036507; doi:10.1016/j.jpsychires.2016.07.013)
Supplement: Supplementary file 1 [file mmc1.docx]

**Supplementary Data**

**Decreased anticipated pleasure correlates with increased salience network resting state functional connectivity in adolescents with depressive symptomatology.**

**Running title**:

Resting-state functional connectivity in adolescents at high risk for depression.

Category:

Regular research article

Ewelina Rzepa MSc, Ciara McCabe* PhD.

School of Psychology and Clinical Language Sciences, University of Reading, UK.

* Corresponding author:

**Dr Ciara McCabe**

Associate Professor in Neuroscience,

School of Psychology and Clinical Language Sciences,

University of Reading,

Reading RG6 6AL,

Tel: +44 118 378 5450

[c.mccabe@reading.ac.uk](mailto:c.mccabe@reading.ac.uk)

Table S1: Mood, energy and affect means and SD for BFS and VAS.

| Measure | | HR; mean (SD) | LR; mean (SD) |
| --- | --- | --- | --- |
| *Before* |  |  |  |
| BFS | | 48.47 (16.69) | 26.4 (18.1) |
| VAS alert | | 5.75 (1.72) | 6.22 (1.99) |
| VAS disgust | | 1.81 (2.55) | .74 (1.39) |
| VAS drowsiness | | 3.36 (2.64) | 2.16 (2.19) |
| VAS anxiety | | 3.5 (2.38) | 1.34 (1.58) |
| VAS happiness | | 5.82 (2.2) | 7.0 (2.31) |
| VAS nausea | | 2.25 (2.83) | 1.02 (1.81) |
| VAS sadness | | 2.32 (2.5) | .75 (1.28) |
| VAS withdrawn | | 3.27 (3.09) | .81 (1.13) |
| VAS faint | | 1.3 (2.36) | .37 (.9) |
| *After* |  |  |  |
| BFS | | 42.53 (13.92) | 31.8 (14.9) |
| VAS alert | | 5.29 (2.39) | 5.44 (2.7) |
| VAS disgust | | 1.3 (1.65) | 2.36 (3.16) |
| VAS drowsiness | | 4.93 (2.69) | 3.8 (3.28) |
| VAS anxiety | | 2.18 (2.25) | .66 (1.13) |
| VAS happiness | | 6.16 (2.01) | 7.46 (1.91) |
| VAS nausea | | 1.4 (1.96) | 2.52 (3.03) |
| VAS sadness | | 1.49 (1.48) | .66 (1.6) |
| VAS withdrawn | | 2.37 (2.04) | .95 (2.23) |
| VAS faint | | 1.78 (2.33) | .89 (.9) |

Table S2. Functional connectivity between seeds and whole brain in healthy control group only.

|  |  | MNI coordinates | |  | | |  |
| --- | --- | --- | --- | --- | --- | --- | --- |
|  |  | X Y | | Z | | | z-score |
| **Left Amygdala** | |  |  |  | | |  |
| pgACC | | -2 | 34 | -2 | | | 8.47 |
| Paracingulate gyrus | | 6 | 52 | 2 | | | 6.42 |
| ACC | | 2 | 34 | 12 | | | 6.1 |
| Lateral Occipital Cortex | | -46 | -76 | 40 | | | 4.02 |
| Lateral Occipital Cortex | | 48 | -60 | 30 | | | 3.82 |
| **Right Amygdala** | |  |  |  | | |  |
| Amygdala | | 22 | -4 | -24 | | | 7.69 |
| Insula | | 42 | -6 | 4 | | | 4.59 |
| Central Opecular Cortex | | 44 | -2 | 6 | | | 4.51 |
| Planum Temporale | | 60 | -10 | 6 | | | 4.29 |
| Hippocampus | | -16 | -10 | -18 | | | 5.54 |
| Amygdala | | -18 | -6 | -16 | | | 5.13 |
| Temporal Pole | | -24 | 6 | -24 | | | 4.84 |
| Brain stem  Inferior Temporal Gyrus  **Left dmPFC** | | 8  50 | -36  -54 | -42  -14 | | | 3.38  3.84 |
| Middle Frontal Gyrus | | -22 | 34 | 26 | | | 7.46 |
| Frontal Pole | | -28 | 46 | 16 | | | 5.35 |
| Paracingulate Gyrus/ACC | | -6 | 32 | 28 | | | 4.59 |
| Middle Frontal Gyrus | | 28 | 30 | 36 | | | 4.68 |
| Frontal Pole | | 32 | 40 | 34 | | | 4.56 |
| Superior Parietal Lobule | | -30 | -42 | 56 | 3.9 | | |
| Precuneus Cortex  Postcentral gyrus | | -12  -30 | -46  -34 | 50  54 | 3.71  3.28 | | |
| **Right dmPFC** | |  |  |  |  | | |
| Frontal Pole | | 18 | 34 | 30 | 7.32 | | |
| Paracingulate Gyrus | | 10 | 36 | 30 | 5.98 | | |
| Superior Frontal Gyrus | | 16 | 32 | 42 | 4.75 | | |
| Lateral Occipital Cortex | | 58 | -60 | 34 | 4.01 | | |
| Cuneal Cortex | | 6 | -70 | 24 | 3.75 | | |
| Precuneus Cortex | | 8 | -58 | 8 | 3.54 | | |
| Paracingulate Gyrus | | -18 | 48 | 4 | | 3.27 | |
| Middle Temporal Gyrus | | 62 | -8 | -16 | | 3.78 | |
| **pgACC** | |  |  |  | |  | |
| Thalamus | | -4 | 0 | -2 | | 8.38 | |
| Thalamus | | 2 | -2 | 0 | | 8.3 | |
| Putamen | | -16 | 8 | -2 | | 4.52 | |
| ACC | | 6 | 42 | 6 | | 4.28 | |
| Frontal Medial Cortex | | -4 | 42 | -12 | | 4.09 | |
| Superior frontal Gyrus | | -18 | 32 | 38 | | 3.73 | |
| Frontal pole | | -16 | 40 | 38 | | 2.85 | |

OFC- orbitofrontal cortex; PCC- posterior cingulate cortex; ACC- anterior cingulate cortex; pgACC-pregenual anterior cingulate cortex
